# Blocking intruders: inducible physico-chemical barriers against plant vascular wilt pathogens

**DOI:** 10.1093/jxb/eraa444

**Published:** 2020-09-25

**Authors:** Anurag Kashyap, Marc Planas-Marquès, Montserrat Capellades, Marc Valls, Núria S Coll

**Affiliations:** 1 Centre for Research in Agricultural Genomics (CSIC-IRTA-UAB-UB), Bellaterra, Spain; 2 Genetics Department, Universitat de Barcelona, Barcelona, Spain; 3 University of Illinois, USA

**Keywords:** Gels, inducible defenses, lignin, physico-chemical barriers, plant-pathogen interactions, structural defenses, suberin, tyloses, vascular pathogens, wilt

## Abstract

Xylem vascular wilt pathogens cause devastating diseases in plants. Proliferation of these pathogens in the xylem causes massive disruption of water and mineral transport, resulting in severe wilting and death of the infected plants. Upon reaching the xylem vascular tissue, these pathogens multiply profusely, spreading vertically within the xylem sap, and horizontally between vessels and to the surrounding tissues. Plant resistance to these pathogens is very complex. One of the most effective defense responses in resistant plants is the formation of physico-chemical barriers in the xylem tissue. Vertical spread within the vessel lumen is restricted by structural barriers, namely, tyloses and gels. Horizontal spread to the apoplast and surrounding healthy vessels and tissues is prevented by vascular coating of the colonized vessels with lignin and suberin. Both vertical and horizontal barriers compartmentalize the pathogen at the infection site and contribute to their elimination. Induction of these defenses are tightly coordinated, both temporally and spatially, to avoid detrimental consequences such as cavitation and embolism. We discuss current knowledge on mechanisms underlying plant-inducible structural barriers against major xylem-colonizing pathogens. This knowledge may be applied to engineer metabolic pathways of vascular coating compounds in specific cells, to produce plants resistant towards xylem colonizers.

## Teaser

An important component of plant resistance towards xylem vascular wilt pathogens is the induction of vertical and horizontal structural barriers, restricting the pathogen to infected vessels, thus avoiding plant wilting.

## Introduction

The plant immune system has been shaped by hundreds of millions of years of interactions with microbial pathogens that are constantly trying to evade or overcome host plant defense reactions. Pathogens that colonize the xylem - a vital component for transporting water and minerals from the roots to the aerial parts - are particularly pernicious, causing major destruction in numerous host plants worldwide (Yadeta and Thomma, 2013). Despite the xylem being nutritionally poor in comparison with other plant tissues, a select group of pathogens comprising a few genera of bacteria, fungi and oomycetes, have evolved to target this plant tissue as a niche for colonization. Proliferation of these wilt pathogens causes massive disruption in the xylem vessels through the production of toxins, exopolysaccharides, hyphal mass, conidia and other pathogen propagules that cause destruction as well as physical occlusion of xylem elements, resulting in severe wilting and plant death (Zaini *et al.*, 2018).

### Invasion strategies used by vascular wilt pathogens

Vascular plant pathogens utilize diverse strategies to get access into the vessels. Vascular wilt pathogenic fungi (*Fusarium oxysporum*, *Verticillium albo-atrum*, *Verticiullium dahliae*, *Ceratocystis fimbriata*, *Ophiostoma novo-ulmi*), oomycetes (*Pythium* spp) or the soil-borne bacterium *Ralstonia solanacearum* invade plant roots and advance inter- or intracellularly through the root cortex to reach the xylem, where they proliferate and spread systemically to aerial plant parts (Bae *et al.*, 2015). In order to get access into the vasculature, these pathogens generally target root extremities and the junction between primary and lateral roots where the epidermal barrier may be compromised, and the endodermis as well as Casparian strip are either not fully differentiated or reoriented by outgrowth of lateral roots (Vasse *et al.*, 1995; Álvarez *et al.*, 2010).

In contrast to these root invaders, many vascular bacteria, such as *Xylella fastidiosa*, are directly inoculated into the xylem by insect vectors that feed on the plant host (Wang *et al.*, 2017). Furthermore, a few species of vascular bacteria reach the xylem tissues via natural plant openings in the aerial parts of the plant, such as leaf hydathodes (*Xanthomonas oryzae* pv. *Oryzae;*Pradhan *et al.*, 2012), flower nectarthodes (*Erwinia amylovora;*Bubán *et al.*, 2003) or stem lenticels (*Pseudomonas syringae pv. actinidiae;*Renzi *et al.*, 2012). These bacteria multiply in intercellular spaces before eventually colonizing the xylem vessels. Moreover, there are vascular wilt pathogens which are transmitted through graft-infected rootstock/scion, as has been reported for the fungus *Ceratocystis fagacearum* on oak trees (Blaedow and Juzwik, 2010), or the bacterium *X. fastidiosa* on pecan (Sanderlin and Melanson, 2006). Another method of infection of vascular wilt pathogens occurs through the use of infected mother plants as a source for clonal propagation. This affects many plant species cultivated in nurseries, such as strawberry plants infected with *Fusarium oxysporum* f. sp. *fragariae* (Pastrana *et al.*, 2019), olive plants infected with *V. dahliae* (Morello *et al.*, 2016), and it is also common in grapevine mother plants that can harbor various vascular wilt fungi (Gramaje *et al.*, 2018).

### Immunity against vascular wilt pathogens

Plants are protected against pathogens by a well-orchestrated immune system (Jones and Dangl, 2006). Pattern-triggered immunity (PTI) is initiated upon recognition of conserved microbial features at the plasma membrane by pattern-recognition receptors (PRRs) in the plant. Effector-triggered immunity (ETI) is activated by recognition of pathogen-secreted effectors via intracellular nucleotide-binding leucine-rich repeat proteins (NLRs). ETI is commonly known as a stronger immune response, while PTI offers durable and broad-spectrum resistance (Katagiri and Tsuda, 2010). Yet, these two layers of immunity operate synergistically, converging on several downstream responses including the oxidative burst, activation of kinase signaling cascades, expression of defense-related genes and accumulation of physico-chemical barriers (Thomma *et al.*, 2011; Ngou *et al.*, 2020, Preprint; Yuan *et al.*, 2020, Preprint).

Although PTI and ETI function in the interactions of plants with vascular pathogens, there are very few cases where these processes have been analyzed specifically in the xylem and surrounding tissues of infected plants. Several recent studies have shown that PTI can be triggered in plants by treatment or over-expression of conserved molecular patterns present in wilt pathogens. For example, an extracellular polysaccharide from the wilt bacterium *R. solanacearum* has been shown to act as a PTI elicitor in resistant tomato, leading to induced expression of defense response genes (Milling *et al.*, 2011; Prakasha *et al.*, 2017). Furthermore, in *Nicotiana benthamiana* and tomato, PTI is triggered upon perception of the COLD SHOCK PROTEIN 22 (csp22) peptide from *R. solanacearum* by the PRR COLD SHOCK PROTEIN RECEPTOR (CORE; Wang *et al.*, 2016). In accordance, treatment of tomato with csp22 conferred increased resistance to *R. solanacearum* in tomato plants. Additionally, transgenic *Arabidopsis thaliana* plants expressing the tomato csp22 receptor (SlCORE) gained the ability to respond to csp22 and were more resistant to *R. solanacearum* infection (Wei *et al.*, 2018). Similarly, *V. dahliae*, possesses two cellulose-degrading glycoside hydrolase family 12 (GH12) proteins, namely VdEG1 and VdEG3, which in *N. benthamiana* are recognized by the PRR complexes LRR-RLP (Leucine Rich Repeat-Receptor-Like Protein)/SOBIR1/BAK1 (SUPPRESSOR OF BAK1-INTERACTING RECEPTOR-LIKE KINASE 1-1/BRASSINOSTEROID-INSENSITIVE 1-ASSOCIATED RECEPTOR KINASE) and LRR-RLKs (Receptor-Like Kinase)/BAK1, respectively, thereby triggering PTI responses (Gui *et al.*, 2017).

Concomitantly, some bacterial wilt pathogens have evolved modifications in the sequences of their conserved patterns, so that they are no longer recognizable by cognate PRRs. This occurs in *X. fastidiosa*, which contains a lipopolysaccharide featuring a masking motif that evades recognition by grapevine (Rapicavoli *et al.*, 2018), or flagellin from *R. solanacearum*, with a polymorphic sequence that avoids perception by many host plants (Wei *et al.*, 2020).

An interesting emerging concept is that roots may be relatively insensitive to most conserved molecular patterns, which may prevent mounting defense responses against commensal organisms in an environment such as the soil, full of microbes. Underlying this phenomenon, it has been recently shown that root PTI is only activated upon local tissue damage (Zhou *et al.*, 2020). This work convincingly shows how localized cell death upregulates PRR expression in the neighboring cells, leading to restricted responsiveness to conserved molecular patterns of root invaders (Zhou *et al.*, 2020).

ETI responses against vascular pathogens have also been documented in the literature. For instance, in the interaction between tomato and the vascular wilt fungus *F. oxysporum* f. sp. *lycopersici*, several R protein-effector pairs have been identified. The NLR I2 perceives the Avr2 effector; I, a receptor-like protein with an extracellular LRR domain (LRR-RLP) perceives Avr1; and I3, a S-receptor-like kinase (SRLK) perceives Avr3 (Houterman *et al.*, 2009; Catanzariti *et al*., 2015, 2017). Interestingly, the *I2* gene is primarily expressed in the xylem vascular tissue, probably partly explaining why the fungus reaches the vasculature before being contained in an incompatible interaction (van der Does *et al.*, 2019; Mes *et al.*, 2000). Similarly, effector AvrFom2 from *F. oxysporum* f. sp. *melonis* is perceived by the NLR Fom-2 in melon (Joobeur *et al.*, 2004). On the other hand, the effector Ave1 from *V. albo-atrum* induces resistance in several plant species, mediated by the receptor-like protein Ve1 (de Jonge *et al.*, 2012; Song *et al.*, 2018).

With respect to bacterial vascular wilt pathogens, the RRS1-R/RPS4 (Resistance to *Ralstonia solanacearum 1*-Recessive/ Resistant to *Pseudomonas syringae 4*) NLR pair in Arabidopsis mediates immunity against *R. solanacearum* carrying the PopP2 effector (Deslandes *et al.*, 2002; Le Roux *et al.*, 2015; Sarris *et al.*, 2015). The rice LRR-RLK protein Xa21 confers broad-spectrum immunity to *X. oryzae* pv. *oryzae*, mediated through recognition of the small protein Ax21 (Park and Ronald, 2012). In addition, the rice NLR Xo1 recognizes the transcription activator-like effector Tal2H from *X. oryzae* pvs. *oryzicola* and *oryzae* (Triplett *et al.*, 2016). Similarly, the NLR Bs4 in tomato perceives the avirulence protein AvrBs4 from *Xanthomonas campestris* pv. *vesicatoria* (Schornack *et al.*, 2004). Furthermore, the Arabidopsis NLR ZAR1 (HOPZ-ACTIVATED RESISTANCE 1) recognizes the *X. campestris* effector AvrAC/XopAC by forming a complex with PBL2 (PROBABLE SERINE/THREONINE-PROTEIN KINASE 2), which acts as a decoy, and RKS1 (RESISTANCE-RELATED kinase 1), a pseudokinase (Wang *et al.*, 2015).

Besides individual examples of *R* genes, quantitative traits have been shown to underscore immunity against xylem colonizers in many different hosts. Stable resistance towards *R. solanacearum* in tomato is controlled by two major quantitative trait loci (QTLs), namely *Bwr*-*6* and *Bwr*-*12*, located in chromosomes six and twelve, respectively, and three minor QTLs, *Bwr-3*, *Bwr-4* and *Bwr-8* (Thoquet *et al.*, 1996; Mangin *et al.*, 1999; Wang *et al*., 2000, 2013; Carmeille *et al.*, 2006). Some of these loci have been shown to be strain and/or environment-specific, and involve an extremely complex genetic basis of resistance. Similarly, QTLs for *Fusarium* wilt resistance have been mapped in several hosts such as chickpea (Sabbavarapu *et al.*, 2013), cotton (Ulloa *et al.*, 2013; Wang *et al.*, 2018), and watermelon (Lambel *et al.*, 2014). QTLs termed RESISTANCE TO FUSARIUM (*RFO1-RFO7*) have been identified in Arabidopsis that provide broad spectrum immunity to multiple *formae speciales* of *F. oxysporum* (Chen *et al.*, 2014). *RFO1* was found to encode a wall-associated kinase-like kinase 22 (WAKL22) and *RFO2* encodes a receptor-like protein (Diener and Ausubel, 2005). Likewise, resistance to *Verticillium* correlates with mapping of QTLs in several hosts such as strawberry (Antanaviciute *et al.*, 2015) and cotton (Palanga *et al.*, 2017). A major QTL conferring resistance to *X. fastidiosa* was identified in a *Vitis arizonica* linkage map and named as ‘Pierce’s disease resistance 1’ (PdR1), which has been introgressed into commercial cultivars (Krivanek *et al.*, 2006). However, the majority of available QTL mapping data for wilt resistance traits lack resolution. This may be partly due to the high sensitivity of wilt development to temperature variation, and also due to the fact that wilt resistance comprises a complex array of multilayered mechanisms involving the coordinated action of different plant tissues or cell types (Bani *et al.*, 2018). Importantly, this extremely complex polygenic and quantitative resistance underscores the structural physico-chemical defense mechanisms induced by vascular pathogens in resistant plants.

### Inducible structural barriers to restrict the spread of vascular wilt pathogens

Inducible structural barriers formed at and around the vasculature upon colonization constitute one of the most important defense components against wilt diseases. If a pathogen manages to reach the xylem, this transport system becomes an excellent channel of inoculum dissemination throughout the plant. As a consequence, plants have evolved effective structural defense mechanisms to prevent vessel colonization or movement between vessels once vascular colonization has occurred (Beckman and Roberts, 1995) Timely formation of these physico-chemical vascular barriers early upon pathogen perception can lead to confinement of the vascular pathogen at the infected vessel, avoiding the spread of wilt diseases (Robb *et al.*, 2007; Zaini *et al.*, 2018; Planas-Marquès *et al.*, 2019).

Some of these structural reinforcements induced by pathogens were already reported in classic botanical studies in the 19th century (Zimmermann, 1979). Moreover, during the 1980s and 1990s, they were intensely studied from anatomical and biochemical points of view, as they were recognized as important components of defense reactions (Newcombe and Robb, 1988; Nicholson, 1992; Niemann, 1994). In trees, the sequential response to confine fungal pathogen progression or tissue damage at the site of infection or injury was initially explained by the CODIT model (compartmentalization of decay in trees; Shigo and Marx, 1977). The model described four “walls” or barriers pre-formed or formed in response to wounding, that restrict pathogen colonization. In particular, wall 1 defined vessel plugging structures formed as a response to vascular wilt pathogen invasion. 

However, most of the research in the field of plant-pathogen interactions started refocusing during the 1990s when Arabidopsis gained momentum as a model species. This led to the identification of striking similarities between plant and animal immune systems, the type III secretion system in bacteria being discovered, and *avr*-*R* gene pairs being identified as a corollary of Flor’s gene-for-gene model (Nishimura and Dangl, 2010). We now think it is time to revisit the role of inducible structural defenses in plant-pathogen interactions. These defenses are extremely important to block progression of the devastating vascular pathogens, and we are far from understanding how these types of defense mechanisms are controlled.

**Fig. 1.**
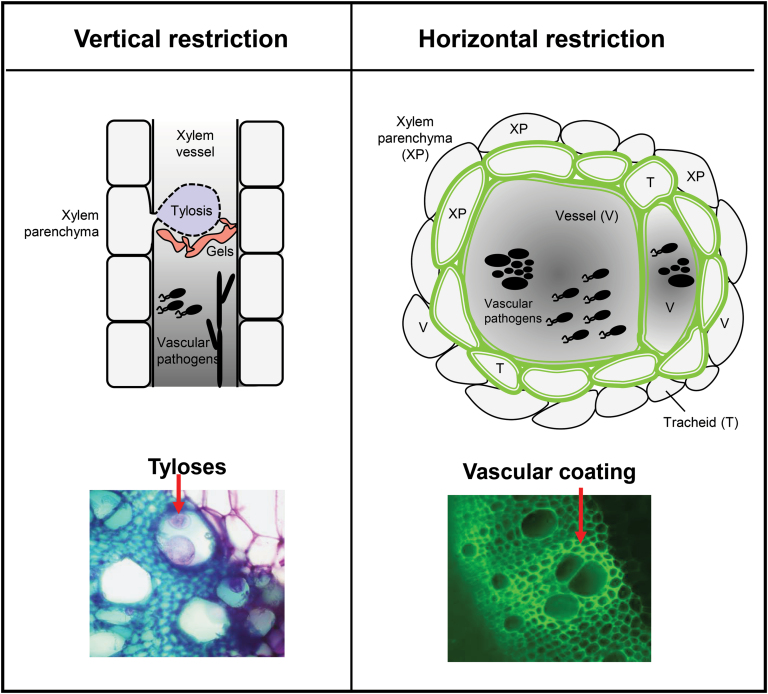
**The two dimensions of plant physico-chemical barriers induced against xylem vascular wilt pathogens.** To counter invasion by xylem vascular wilt pathogens, resistant plants induce two-dimensional physico-chemical defenses that restrict vertical and horizontal movement of the pathogen. Vertical spread within the vessel lumen is mainly restricted by tyloses and gels (left). In contrast, horizontal spread of the pathogen to surrounding healthy vessels is prevented by reinforcement of the walls of colonized vessels (V) and the surrounding xylem parenchyma (XP) and tracheids (T), through vascular coating with mainly lignin and suberin (shown as a green color in the diagram). Synchronized formation of vertical and horizontal barriers early after pathogen invasion results in compartmentalization of the pathogen inoculum at the site of infection, thereby preventing wilt, and constitute a major component of resistance. To visualize tyloses and vascular coating (panels below), tomato root cross-sections were obtained after *R. solanacearum* soil soak inoculation, and fixed in 70% ethanol. For tyloses, cross-sections were stained with 0.1% toluidine blue and observed using a Leica DM6B-Z microscope under bright field conditions, and images were recorded through a MC190-HD-0518131623 camera. To visualize phenolic vascular coating, the cross-sections were illuminated by UV using a Leica DM6B-Z microscope, and the auto-florescence emitted from phenolic deposits was observed using a HC PL FLUOTAR objective. Images were captured using a Leica-DFC9000GT-VSC07341 camera. In the left panel, the arrow points towards *R. solanacearum-*induced formation of tyloses inside vessel lumen, which appear pink to violet color upon staining with 0.1% toluidine blue. In the right panel, the arrow points towards *R. solanacearum-*induced auto-fluorescence emitted from phenolics, deposited in the walls of vessels and the surrounding tracheids and parenchyma cells. Scale bar=120 µm.

In this review, we summarize how inducible vascular structures compartmentalize the vascular wilt pathogens leading to resistance. For this, we classify inducible structural barriers using a bi-dimensional perspective. We define a vertical and a horizontal component of resistance as those structures that restrict the vertical and horizontal movement of the pathogen, respectively, within the xylem vascular tissue (Fig. 1, Table 1). Furthermore, we review current knowledge on mechanisms underlying plant-inducible structural defenses against major xylem-colonizing pathogens, highlighting their correlation with biochemical changes and genetic interactions. Finally, we discuss future perspectives in the study of inducible vascular structural defenses, taking into account recent technological advances and how this may be translated into increasing resistance to vascular wilt pathogens in the field. Such physico-chemical defense responses are key traits desired for effective management of vascular wilt pathogens (Ferreira *et al.*, 2017).

**Table 1.** List of plant pathosystems in which (I) vertical or (II) horizontal restriction of pathogen movement inside the plant has been shown.

I.	Vertical restriction		
**Structure**	**Host**	**Pathogen**	**Reference**
**Tylose formation**	Banana	*Fusarium oxysporum* f.sp. *cubense*	(Vander Molen *et al.*, 1987)
	Butternut	*Ophiognomonia clavigignenti-juglandacearum*	(Rioux *et al.*, 2018)
	Cotton	*Fusarium oxysporum* f. sp. *vasinfectum*	(Shi *et al.*, 1991)
	Cucurbits	*Fusarium oxysporum f. sp. melonis*	(Seo and Kim, 2017)
	Elm	*Ophiostoma novo-ulmi*	(Plichta *et al.*, 2016)
	Grapevine	*Xylella fastidiosa*	(Sun *et al.*, 2013)
		*Phaeomoniella chlamydospora*	(Pouzoulet *et al.*, 2013)
	Potato	*Rasltonia solanacearum*	(Ferreira *et al.*, 2017)
	Tomato	*Rasltonia solanacearum*	(Grimault *et al.*, 1994)
		*Verticillium albo-atrum*	(Hutson and Smith, 1980)
		*Verticillium dahliae*	(Tjamos and Smith, 1975)
		*Fusarium oxysporum* f. sp. *lycopersici*	(Hutson and Smith, 1980)
**Gel deposition**	Banana	*Fusarium oxysporum* f.sp. *cubense*	(Vander Molen *et al.*, 1987)
	Butternut	*Ophiognomonia clavigignenti-juglandacearum*	(Rioux *et al.*, 2018)
	Carnation	*Fusarium oxysporum* f.sp. *dianthi*	(Baayen and Elgersma, 1985)
	Elm	*Ophiostoma novo-ulmi*	(Plichta *et al.*, 2016)
	Grapevine	*Xylella fastidiosa*	(Sun *et al.*, 2013)
	Pea	*Fusarium oxysporum* f. sp. *pisi*	(Bishop and Cooper, 1983)
	Plane tree	*Ceratocystis fimbriata f. sp platani*	(Clérivet *et al.*, 2000)
	Tomato	*Rasltonia solanacearum*	(Grimault *et al.*, 1994; Kim *et al.*, 2016)
		*Verticillium albo-atrum*	(Hutson and Smith, 1980)
		*Fusarium oxysporum* f. sp. *lycopersici*	(Hutson and Smith, 1980)
**II.**	**Horizontal restriction**		
**Structure**	**Host**	**Pathogen**	**Reference**
**Lignin deposition**	Banana	*Fusarium oxysporum f. sp. cubense*	(De Ascensao and Dubery, 2000)
	Cotton	*Verticillium dahliae*	(Xu *et al.*, 2011; Bu *et al.*, 2014)
	Dutch elm	*Ophiostoma novo-ulmi*	(Martin *et al.*, 2007)
	Flax	*Fusarium oxysporum f. sp. lini*	(Galindo-González and Deyholos, 2016)
	Oilseed rape	*Verticillium longisporum*	(Eynck *et al.*, 2009)
	Olive	*Xylella fastidiosa*	(Sabella *et al.*, 2018)
	Pepper	*Verticillium dahliae*	(Novo *et al.*, 2017)
	Potato	*Rasltonia solanacearum*	(Ferreira *et al.*, 2017)
	Tomato	*Verticillium dahliae*	(Street *et al.*, 1986; Hu *et al.*, 2019)
		*Rasltonia solanacearum*	(Ishihara *et al.*, 2012; Ferreira *et al.*, 2017)
**Suberin deposition**	Alfalfa	*Verticillium albo-atrum*	(Newcombe and Robb, 1988)
	Butternut	*Ophiognomonia clavigignenti-juglandacearum*	(Rioux *et al.*, 2018)
	Dutch elm	*Ophiostoma novo-ulmi*	(Martín *et al.*, 2008)
	Grapevine	*Phaeomoniella chlamydospora*	(Pouzoulet *et al.*, 2013)
	Tomato	*Verticillium albo-atrum*	(Street *et al.*, 1986; Robb *et al.*, 1991)

### Vertical restriction of vascular colonization

Once they reach the xylem, different lumen occlusions constitute vertical barriers to the anti-gravitational movement of vascular pathogens. Vascular occlusion is an effective means of slowing down vertical progression of the pathogen, or even confining it to the infection site, preventing systemic infection. The most prominent of these occlusions are tyloses and gels, which we will review in this section, focusing on the aspects related to plant defense. As an important defense strategy by vascular pathogens, mechanisms to evade or subvert vertical occlusions and secure colonization have evolved on the pathogen side, as part of the evolutionary arms race. Inhibition of vertical occlusions has been reported for various wilt fungi, although the mechanisms for this virulence mechanism are not fully understood (Beckman and Roberts, 1995; Pouzoulet *et al.*, 2017).

### Formation of tyloses

Tyloses (singular tylosis) are balloon-like overgrowths of the protoplast of adjacent living parenchyma cells that protrude into xylem vessels through its pits (Fig. 1; Bonsen and Kucera, 1990). Tyloses are considered inducible defense structures against xylem intruders because their formation can prevent spreading of the pathogen and also protect healthy parts of the plant by blocking the infected vessels (Leśniewska *et al.*, 2017). However, formation of tyloses is not only linked to pathogen attack, as they can be induced by several other environmental stimuli, such as pruning, wounding, flooding and frost (Davison and Tay, 1985; Cochard and Tyree, 1990; Sun *et al.*, 2007; De Micco *et al.*, 2016). The process of tylosis formation is tightly controlled and has certain commonalities with regular cell enlargement (Bishop and Cooper, 1984). Hormones such as auxin, ethylene and jasmonate seem to have a prominent role in the formation of these structures (Vander Molen *et al.*, 1987; Leśniewska *et al.*, 2017).

The formation of tyloses in response to pathogen attack has been extensively reported (Table 1; Cooper and Williams, 2004; Sun *et al.*, 2013; Ferreira *et al.*, 2017; Rioux *et al.*, 2018). Restricted formation of tyloses has been observed and specifically induced in the infected vessels of resistant tomato and potato varieties, effectively restricting *R. solanacearum* to the infected vascular bundles (Grimault *et al.*, 1994; Ferreira *et al.*, 2017). Such specific induction was not observed in susceptible tomato cultivars infected with *R. solanacearum*, where the formation of tyloses appeared delayed and less focused, with numerous non-colonized vessels occluded by tyloses, and pathogen growth unrestricted (Grimault *et al.*, 1994). Tylosis formation in resistant tomato cultivars has also been observed upon inoculation with the pathogenic fungus *F. oxysporum* f. sp. *lycopersici* and *V. albo-atrum* (Hutson and Smith, 1980). Similarly, in a banana cultivar resistant to race 1 of *F. oxysporum* f. sp. *cubense*, tylosis initially appeared as early as within two days post-inoculation in the lumen of xylem vessels of the root (Vander Molen *et al.*, 1987).

In addition to acting as structural barriers, tyloses can act as storage organs of antimicrobial compounds. Although they are predominantly composed of pectic substances (Rioux *et al.*, 1998; Eynck *et al.*, 2009), fungicidal compounds such as elemental sulfur have been detected in tyloses of tomato lines resistant to *V. albo-atrum*, which was thought to inhibit spore germination (Williams *et al.*, 2002). In addition, a fully developed tylosis also develops a lignified and/or suberized cell wall, which may protect the exposed cell from the xylem pathogen (Pouzoulet *et al.*, 2013).

However, the formation of tyloses is not always linked to resistance. For instance, a tomato variety susceptible to *V. albo-atrum* formed a few miniature tyloses in response to infection that the fungal hyphae were able to easily surpass within the vessel (Tjamos and Smith, 1975). More important than the quantity or frequency of tyloses formed, the successful restriction of a vascular pathogen depends on synchronization and specificity of tylose production to pathogen-colonized vessels (Bishop and Cooper, 1984). Sun *et al.*, (2013) reported tyloses as the predominant type of occlusion that formed in grapevines with differing resistance to *X. fastidiosa*. Excessive tylosis formation in response to *X. fastidiosa* infection in the susceptible grapevine cultivar led to heavy blockage of vessels and development of wilting symptoms, and did not significantly affect pathogen spread. In contrast, in resistant grapevines, tylosis development was specific and mainly limited to a few internodes close to the point of inoculation, impacting less of the vessels and indicating that timing and localization are key (Sun *et al.*, 2013). Interestingly, the anatomy of xylem vessels also plays a role in augmenting pathogen compartmentalization by tyloses. Cultivars of grapevine having larger vessel size are known to be susceptible to vascular wilt pathogens such as *X. fastidiosa*, *Eutypa lata, Phaeoacremonium aleophilum, Phaeomoniella chlamydospora, Diplodia seriata,* and *Neofusicoccum parvum* (Pouzoulet *et al*., 2017, 2019, 2020; Deyett *et al.*, 2019). Likewise, Dutch elm cultivars having larger vessel diameters are susceptible to *Ophiostoma novo-ulmi* (Pouzoulet *et al.*, 2014; Venturas *et al.*, 2014). Across the grapevine genotypes, it has been observed that the extent of *P. chlamydospora* compartmentalization is a function of the diameter of the host xylem vessels. Genotypes with increased number of xylem vessels above 100 µm in diameter resulted in increased infection of host tissue (Pouzoulet *et al.*, 2020). Though numerous tyloses are formed in large vessels of susceptible grapevine cultivars in response to the wilt pathogen *P. chlamydospora,* the compartmentalization process is not as efficient as in narrow diameter vessels, due to the presence of large escape routes (Pouzoulet *et al.*, 2017).

### Deposition of gels

Deposition of electrodense material corresponding to gels or gums in the lumen of the xylem vessels is another feature commonly observed during xylem invasion by vascular pathogens, and acts as one of the multiple factors that contribute to induced structural defense (Vander Molen *et al.*, 1977). Besides its role in immunity, these occluding structures also form in response to other stimuli such as wounding or aging (Ratnayake *et al.*, 2013).

Gels are commonly secreted by xylem parenchyma cells and they are transported across pit membranes into vessel elements (Fig. 1; Bishop and Cooper, 1984). Tyloses have also been observed to secrete gels into the lumen of vessels, thereby multiplying the clogging effect (Bonsen and Kucera, 1990; Rioux *et al.*, 1998). Gels appear fibrillar, forming thin networks of varying electron density that ultimately fill and clog the vessel lumen (Sun *et al.*, 2008). Gels initially appear as translucent fibres arising from several places along lateral walls of vessels, and later form a continuous layer with wavy edges toward the vessel lumen. Subsequently, gels turn yellow and are interspersed with small particles, coinciding with vessel occlusions (Sun *et al.*, 2008). Although the main component of gels are pectic substances such as partially esterified pectic polysaccharides (Rioux *et al.*, 1998; Clérivet *et al.*, 2000), they may also accumulate antimicrobial compounds such as elemental sulfur and phytoalexins (Cooper and Williams, 2004; Sun *et al.*, 2008). Furthermore, these gels are strengthened by deposition of lignin and other phenolic compounds, which make these plugs strong physical barriers (Kpemoua *et al.*, 1996; Rioux *et al.*, 1998).

Formation of vascular gels is considered an important part of resistance towards several wilt diseases (Table 1). For example, *F. oxysporum* f. sp. *dianthi* colonization is restricted due to the formation of gels in the vascular lumen of carnation plants (Baayen and Elgersma, 1985). Moreover, in most resistant cultivars these gels are often observed together with tyloses (Vander Molen *et al.*, 1987; Grimault *et al.*, 1994). In banana plants resistant to *F. oxysporum* f. sp *cubense,* formation of vascular occlusions including both gels and tyloses have been observed (Vander Molen *et al.*, 1977). Gel formation in the xylem lumen is also a trait of tomato cultivars resistant towards the vascular bacterium *R. solanacearum* (Grimault *et al.*, 1994). In other cases, such as pea plants resistant to *F. oxysporum* f. sp. *pisi*, vascular gels, but not tyloses, are observed after infection (Bishop and Cooper, 1984).

### Horizontal restriction of vascular colonization

**Fig. 2.**
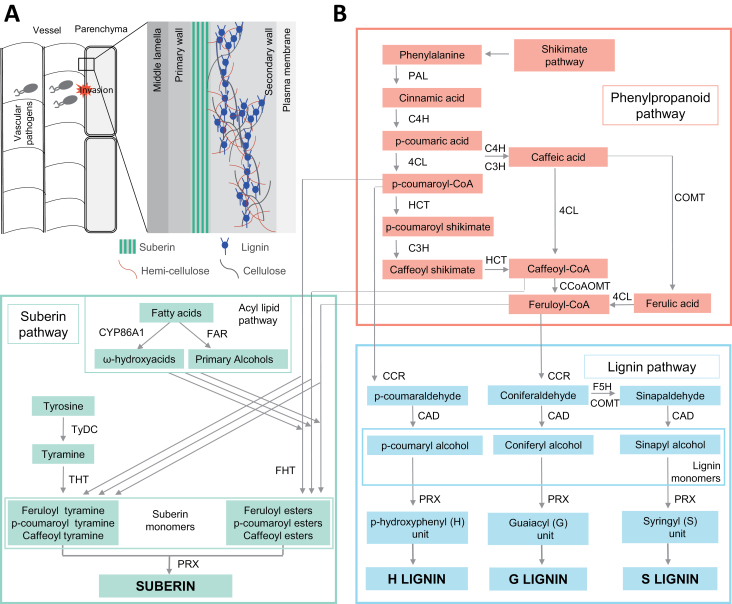
**Lignin and suberin have a major role in vascular coating induced by xylem vascular wilt pathogens.** A. Schematic structure of reinforced cell walls of xylem vessels and parenchyma cells in resistant plants upon infection with xylem vascular wilt pathogens. B. The phenypropanoid pathway provides precursors for both lignin and suberin biosynthesis. Phenylalanine, derived from the shikimate pathway, undergoes several enzymatic reactions as part of the phenylproanoid pathway. The resulting precursors yield the monolignols *p*-coumaryl alcohol, coniferyl alcohol and sinapyl alcohol, which are the building blocks of lignin. In parallel, the phenypropanoid metabolites feruloyl-CoA, caffeoyl-CoA, and *p*-coumaroyl-CoA bifurcate into the suberin pathway. In the suberin pathway, these metabolites can be conjugated to aromatic amine compounds such as tyramine by the action of THT, or can be linked to aliphatic compounds by the action of FHT, to yield suberin monomers. Lignin and suberin monomers are then transported to the cell wall, where they are subsequently polymerized into the reinforcing matrices that constitute vascular coating structures. Abbreviations: PAL: phenylalanine ammonia–lyase; C4H: cinnamate–4–hydroxylase; C3H: coumarate 3-hydroxylase; 4CL: 4–coumarate–CoA ligase; HCT: hydroxycinnamoyl–CoA shikimate/quinate hydroxycinnamoyl transferase; COMT: caffeic acid 3-O-methyltransferase; CCOMT: caffeoyl CoA 3-O-methyltransferase; CCR: cinnamoyl CoA reductase; CAD: cinnamoyl alcohol dehydrogenase; PRX: peroxidase; CYP86A1: fatty acid cytochrome P450 oxidases; FAR: fatty acyl-CoA reductase; TyDC: tyrosine decarboxylase; FHT: feruloyl transferase; THT: tyramine hydroxycinnamoyl transferase.

The mechanisms mentioned above are part of host responses that restrict vertical movement of vascular pathogens to healthy regions of the host. In addition, resistant plants can often develop a protective vascular coating upon invasion by vascular pathogens, posing a horizontal barrier to further colonization of adjacent healthy tissues. Vascular coating involves physico-chemical structural modifications in the cell walls of xylem tissues that result in confinement of the pathogen to the infected vessels (Figs 1, 2A).

Xylem pits are the primary routes of vessel-to-vessel and vessel-to-parenchyma cell water transport. Pits are covered by a pit membrane, which is impermeable to particulate matter like bacteria and other pathogens (Choat *et al.*, 2008). For a pathogen to achieve successful horizontal transfer into vessels, it has to either form openings in vessel walls or degrade pit membranes, thereby reaching the adjacent parenchyma cells and vessels (Nakaho *et al.*, 2000). To avoid the breach by pathogens, resistant plants with altered composition and structure of homogalacturonans (HGs) and xyloglucans (XyGs) in pit membranes have evolved; however, these compounds are potential targets of the pathogen’s cell wall degrading enzymes. Grapevine genotypes resistant to *X. fastidiosa* lacked fucosylated XyGs and weakly methylesterified HGs (ME-HGs), and contained a small amount of heavily ME-HGs. In contrast, pit membranes of susceptible genotypes all had substantial amounts of fucosylated XyGs and weakly ME-HGs, but lacked heavily ME-HGs (Sun *et al.*, 2011).

In addition, reinforcement occurs at vessel walls, parenchyma cells and pit membranes, to confine the spread of vascular pathogens. Ultra-microscopic studies showed that the pit membranes, as well as vessels walls and parenchyma cells, form a conspicuously thick coating in the form of an electron dense amorphous layer, as part of the defense response against vascular pathogens (Street *et al.*, 1986; Benhamou, 1995; Daayf *et al.*, 1997; Nakaho *et al.*, 2000; Araujo *et al.*, 2014). Such reinforcement acts to limit the horizontal movement of the pathogen from the protoxylem or the primary xylem to the surrounding cells (Street *et al.*, 1986; Benhamou, 1995; Daayf *et al.*, 1997; Nakaho *et al.*, 2000; Araujo *et al.*, 2014). Besides, its deposition acts as a shield against pathogen-derived metabolites such as toxins and enzymes, and makes water and nutrients inaccessible for pathogens, thereby impeding their growth (Araujo *et al.*, 2014).

Importantly, the timing of synthesis of the vascular coating plays a crucial role in immunity. Even if vascular coating can be observed in response to vascular pathogens in susceptible plants, these structures form at late time points, compared with their induction in resistant plants (Shi *et al.*, 1991; Daayf *et al.*, 1997). The specific composition of vascular deposits varies depending on the particular host-pathogen interaction. However, phenolics are the most important compounds, as they act as building blocks of the secondary cell wall, and they also have direct antimicrobial activity (Eynck *et al.*, 2009). Among the phenolic polymers constituting vascular coating structures, the principal players are lignin and suberin, described in further detail below. We also outline the role of callose, a non-phenolic compound that plays an important role in the formation of horizontal vascular barriers in certain interactions.

### Deposition of lignin

Lignin is a complex phenolic polymer that constitutes a major component of secondary cell walls in vascular plants. Lignin imparts strength to secondary cell walls, being deposited in spaces between cellulose, hemicellulose and pectin (Kang *et al.*, 2019; Fig. 2A). The building blocks of the lignin polymer are monolignols, synthesized from phenylalanine via the phenylpropanoid pathway, where numerous enzymes are involved (Fig. 2B). Monolignols are then transported from the cytosol into the apoplast, where they are polymerized to lignin units by the oxidative activities of laccases and peroxidases.

Lignin is a particularly important element in cell walls of xylem tissue cells, not only because of its structural function, but also because it facilitates water retention in vascular bundles due to its hydrophobic nature. In addition, lignin is resistant to biodegradation and acts as a potent structural barrier against pathogens. Although lignin is an integral part of pre-existing structural barriers in plants, its deposition can also be induced in the xylem upon pathogen attack to form additional reinforcements that further restrict pathogen colonization. This inducible deposition of lignin as a vascular coating can be an important component of resistance towards certain pathogens (Table 1).

Restriction of pathogen colonization by lignin has been elegantly shown using a non-vascular pathogen, the leaf-infecting bacterium *Pseudomonas syringae* pv *tomato* (*Pto*) on *Arabidopsis thaliana* (Lee *et al.*, 2019). It was shown that infection with either virulent or avirulent strains of the pathogen induced localized lignification at the site of pathogen attack. However, lignin deposition was more conspicuous upon avirulent *Pto* recognition, leading to confinement of the pathogen, and restricting hypersensitive response cell death to the infection site. In contrast, virulent strains could overcome induced lignification, as the deposition in this case was milder, leading to unrestricted disease progression.

Interestingly, Casparian strip membrane domain proteins (CASPs), were shown to be involved in pathogen-induced lignin deposition (Lee *et al.*, 2019). The Casparian strip acts as a diffusion barrier that controls the uptake of water and molecules from the soil into the water-conducting tissues, and prevents the entry of pathogens and other harmful substances. Both the Casparian strip and the above-mentioned pathogen-induced lignification mechanism involve lignin-containing structures that function as anti-pathogenic physical barriers, serving parallel functions. In addition to lignin, suberin (discussed in detail later) is a chief component of the Casparian strip (Doblas *et al.*, 2017). Recent findings show that the Casparian strip possesses a barrier surveillance pathway comprised of the receptor-like cytoplasmic kinase SCHENGEN1 and the LRR-RLK SCHENGEN3, hence bearing a striking resemblance to signaling pathways for perception of pathogen-associated molecular patterns (Alassimone *et al.*, 2016; Fujita *et al.*, 2020). These receptors in the Casparian strip domain interact with peptides (CASPARIAN STRIP INTEGRITY FACTORS, CIF1/2) expressed in the stele, leading to Casparian strip formation. This spatial separation of receptor and ligand constitutes a surveillance system where interaction stops after effective sealing by the strip, but any breach in the barrier leads to re-interaction and further strengthening (Doblas *et al.*, 2017).

Lignin deposits seem to also play a crucial role in spatial growth restriction of vascular pathogens. There are several examples showing a pronounced transcriptional upregulation of genes involved in lignin biosynthesis in resistant plants following infection with various vascular pathogens (Table 1). This occurs, for example, in cotton after *V. dahliae* infection, flax after infection with *F. oxysporum*, tomato after infection with *R. solanacearum*, or olive tree after *X. fastidiosa* infection (Xu *et al.*, 2011; Ishihara *et al.*, 2012; Galindo-González and Deyholos, 2016; Sabella *et al.*, 2018). Furthermore, there are several examples showing that resistance/tolerance to vascular pathogens is accompanied by an increase in lignin content and enhanced cell wall lignification upon infection. This includes examples of different pathosystems such as *V. dahliae—pepper*, *O. novo-ulmi—Ulmus minor*, *V. longisporum—*rapeseed, *F. oxysporum—*banana or tomato, and *R. solanacearum—*potato (Street *et al.*, 1986; De Ascensao and Dubery, 2000; Martín *et al.*, 2005; Martin *et al.*, 2007; Eynck *et al.*, 2009; Ferreira *et al.*, 2017; Novo *et al.*, 2017).

Nevertheless, the mechanisms that orchestrate timely and effective induction of lignin deposition in resistant plants upon vascular colonization remain vastly unknown. Some progress has been made using cotton plants infected with *V. dahliae.* In this pathosystem, two proteins potentially regulating *V. dahliae*-induced vascular lignin deposition have been identified. GhUMC1, a copper-binding protein, is involved in resistance mediated by lignin deposition and jasmonate signaling (Zhu *et al.*, 2018)*. GhUMC1* knock-down plants are more susceptible to the pathogen, and lignin vascular coating is drastically reduced. On the other hand, the proline-rich protein GhHyPRP1 acts as a negative regulator of defense against *V. dahlia*, and was shown to induce lignin deposition (Zhu *et al.*, 2018). In accordance, *GhHyPRP1* knock-down plants displayed more lignin deposition upon infection and were more resistant to the pathogen. Interestingly, PevD1, a *V. dahliae* secreted protein, has been shown to activate defenses in cotton, triggering the expression of phenylpropanoid genes and lignin accumulation (Bu *et al.*, 2014). This may indicate that PevD1 acts as an avirulence effector in cotton triggering a defense reaction that includes lignin deposition in the vasculature, which may lead to pathogen confinement into infected vessels. It remains to be determined whether the differential lignin deposition phenotypes observed in resistant cultivars is a direct consequence of pathogen effector recognition via its secreted effectors, and if so, to what degree the mechanisms are conserved between different pathosystems. Moreover, the molecular players involved also need to be identified.

### Deposition of suberin

Suberin is a heteropolymer that deposits as a poly-lamellar structure between the plasma membrane and the cell wall, forming a hydrophobic protective barrier (Fig. 2A). Suberin is deposited in specialized tissues such as root and tuber epidermis, root endodermis, and seed coats. In addition, suberin is formed in response to several stresses such as wounding, salt injury and pathogen attack (Dixon and Paiva, 1995; Bernards, 2002). Besides providing strength to the cell wall, suberin prevents water loss and pathogen entry by sealing off the layer of suberized cells.

Suberin consists of a polyphenolic and a polyaliphatic domain. The polyphenolic domain is predominantly formed by esters and amides of ferulic acid, as well as other hydroxycinnamic acids, such as caffeic acid and *p*-coumaric acid (Negrel *et al.*, 1995; Lashbrooke *et al.*, 2016; Woolfson *et al.*, 2011; Woolfson, 2018). The aliphatic domain consists of a glycerol-based fatty acid-derived polyester comprised primarily of ω-hydroxyacids, α, ω-dicarboxylic acids, fatty alcohols, and small amounts of hydroxycinnamic acids (mainly alkyl ferulates; Beisson *et al.*, 2012). Ferulic esters composed of ferulic acid esterified to fatty acids are considered as one of the monomers of suberin polymer (Negrel *et al.*, 1995).

Significant progress elucidating suberin biosynthesis has been achieved in the last two decades using molecular genetics approaches, especially in the model species *A. thaliana* and in potato tuber periderm (Ranathunge *et al.*, 2011). Suberin shares the phenypropanoid pathway with lignin (Fig. 2B). This pathway provides the precursors for its polyphenolic domain, which are used by downstream suberin-specific enzymes such as tyramine N-feruloyltransferase (THT) and feruloyl trasferase (FHT; Fig. 2B; Serra *et al.*, 2010; Woolfson, 2018; Woolfson *et al.*, 2011). Similarly, several genes involved in the aliphatic metabolism of suberin have been described, such as fatty acid cytochrome P450 oxidases (CYP86A1), fatty acyl-CoA reductase (FARs), β-ketoacyl-CoA synthases (KCS2/Daisy and KCS20), glycerol-3-phosphate acyltransferase5 (GPAT5), as well as *ATP-BINDING CASSETTE G* (*ABCG*) genes involved in the delivery of suberin monomers to the site of suberization (*ABCG2*, *ABCG6*, and *ABCG20*; Beisson *et al.*, 2007; Höfer *et al.*, 2008; Franke *et al.*, 2009; Vishwanath *et al.*, 2013; Yadav *et al.*, 2014). In addition, a few upstream regulators of the suberin biosynthetic pathway have been identified in *A. thaliana*, such as the MYB transcription factors MYB41, MYB107 and SUBERMAN (MYB39; Kosma *et al.*, 2014; Gou *et al.*, 2017; Cohen *et al.*, 2020).

Suberin reinforcement in the xylem vascular tissue has been long recognized as a potent barrier to colonization by pathogens (Robb *et al.*, 1991). There are numerous studies reporting that suberin deposition in the xylem tissue upon infection contributes to resistance (Table 1). For example, vascular deposits have been observed after infection with *V. albo-atrum* in resistant tomato and alfalfa (Street *et al.*, 1986; Newcombe and Robb, 1988; Robb *et al.*, 1991). Similarly, induction of suberin deposition is an important line of defense in *Ulmus minor* against *O. novo-ulmi* (Martín *et al.*, 2008). Interestingly, exogenous application of phenolic compounds further increased resistance of trees to this pathogen through formation of suberin-like compounds in xylem tissues (Martín *et al.*, 2008). Another example of xylem tissue suberization induced by a vascular pathogen is provided by the histological characterization of grapevine infection by the wilt fungus *Phaeomoniella chlamydospora* (Pouzoulet *et al*., 2013, 2017). It was shown that deposition of suberin in paravascular parenchyma cells is an effective barrier against horizontal *P. chlamydospora* colonization from one vessel to the adjacent vessel. Suberin was also shown to form deposits in tyloses induced by infection with this pathogen (Pouzoulet *et al.*, 2013). Interestingly, inhibition of tylose formation in grapevine by *P. chlamydospora* led to vascular coating of surrounding parenchyma cells with phenolic compounds, including suberin (Pouzoulet *et al.*, 2017). This indicates that sequential/superimposed defense mechanisms are in place to restrict pathogen progression to the infection site, once it has reached the vasculature.

Although suberin deposition seems to be an important component of defense responses against vascular pathogens, very little is known about its regulation. Similar to lignin, the effectiveness of vascular suberization as a structural barrier against horizontal colonization by vascular pathogens largely depends on the spatio-temporal control of its deposition, i.e. formation of suberin deposits early after pathogen detection at the site of vascular invasion. The phytohormones abscisic acid (ABA) and ethylene have both been shown to regulate suberization (Soliday *et al.*, 1978; Cottle and Kolattukudy, 1982; Barberon *et al.*, 2016). Since these two hormones are involved in defense against various vascular pathogens, a possibility exists that pathogen-induced vascular suberization correlates with an increase of hormone concentrations during immune responses. However, the mechanistic links between these hormonal pathways and suberin biosynthesis during pathogen-triggered suberin vascular coating remain to be established.

### Deposition of callose

Callose is a linear amorphous cell wall polysaccharide formed by hundreds of glucose units linked by β-1,3 glucosidic bonds (Stone, 2009). This homopolysaccharide is synthesized from uridine diphosphate glucose by callose synthases (also known as CalS or GSL for glucan synthase-like), large multisubunit complexes at the plasma membrane (Ellinger and Voigt, 2014). Callose is not a particularly abundant polymer in the cell wall, but has very relevant regulatory roles in development, plasmodesmata function, as well as in immunity (Schneider *et al.*, 2016). Pathogen-induced callose deposition has been shown to be localized to callosic papillae, providing structural defense against various pathogens (Schneider *et al.*, 2016). In addition, callose can constitute a matrix for accumulation of antimicrobial compounds, thereby providing targeted delivery of chemical defenses at the sites of pathogen attack (Luna *et al.*, 2011).

In the vasculature, callose has also been shown to act as a structural barrier against fungal wilt pathogens, restricting their horizontal vessel-to-vessel movement. Tomato plants resistant to *F. oxysporum* f. sp. *lycopersici* form callose deposits in paravascular parenchyma cells and at pit membranes in response to infection by this pathogen (Beckman *et al.*, 1982; Mueller and Beckman, 1988). In addition, application of the microbe-associated molecular pattern chitosan, a derivative of chitin, restricts colonization of *F. oxysporum f.sp. lycopersici* by inducing a vascular coating composed of callose and phenolic compounds (Benhamou *et al.*, 1994). Furthermore, cotton roots infected with *V. dahliae* showed reinforcement with callose deposits (Daayf *et al.*, 1997). In contrast, infection by the bacterial wilt pathogen *R. solanacearum* caused deposition of callose in both tolerant and susceptible potato plants, indicating that in this interaction callose may not be as important for resistance towards the pathogen (Ferreira *et al.*, 2017). Additional research is needed to clarify the precise role and regulation of callose as a structural defense mechanism induced upon perception of vascular wilt pathogens.

## Concluding remarks and future prospects

During the last few decades, much evidence has accumulated showing the importance of physico-chemical barriers as a crucial component of resistance towards xylem vascular pathogens. From all the research until now in this field, it becomes clear that the localization and timing of formation of these vascular structures is key for their effectiveness as barriers for pathogen confinement. Resistant plants are able to form vertical and horizontal barriers quickly upon pathogen invasion of the vasculature, confining them to infected vessels and avoiding spread to the rest of the plant. In susceptible plants, formation of the same vascular structures is observed, but not targeted to infected vessels, and later in time, once the pathogen has spread throughout the plant they have no effect on disease progression. However, the mechanisms regulating the spatial and temporal formation of vascular structures leading to effective pathogen confinement, and their precise composition, are old questions that remain unanswered. In fact, it remains unclear as to how vascular wilt pathogens are perceived at the vasculature, and how this perception is transduced into timely and restricted formation of structural defenses. Since effective mechanisms of resistance are very much sought after in breeding programs, in the coming years it will be important to make an effort to advance knowledge in this area, even though inducible structural defenses are governed by complex polygenic traits.

Major technological advances in the last few years have placed plant molecular biologists in a privileged position to make significant advances. Of particularly relevance is the CRISPR-Cas9 technology, which has proven extremely efficient for Solanaceous crops such as tomato, pepper and eggplant, which are severely affected by wilt diseases caused by xylem vascular pathogens (Hu *et al.*, 2019; Li *et al.*, 2019; Wang *et al.*, 2019). Importantly, an array of technologies have emerged that allow the study of specific processes in a cell, or in a tissue-specific manner. These techniques will become instrumental in the study of plant-pathogen interactions, which constitute a localized phenomenon by its very nature. This is particularly the case for colonization of vascular cells by vascular pathogens, since the ability to confine the invading agent is a key feature of resistant plants. With the advent of single-cell technologies it will be possible to attain astonishing resolution when investigating the processes occurring at infected cells and surrounding areas. For instance, RNA sequencing of laser-dissected areas or single cells allows profiling of the transcriptomic landscape after infection at relevant sites. In turn, this will allow the identification of marker genes associated with the formation of structural defenses and the subsequent generation of transgenic marker lines, to be able to track relevant cells/tissues at early time points after infection for their analysis. In addition, extremely sensitive analytical techniques have been developed in recent years that allow the identification and quantification of proteins, small molecules and metabolites, and the interactions between them. This includes Raman spectroscopy or MALDI (matrix-assisted laser desorption ionization) spectrometry imaging, both of which can be extremely useful in zonal responses such as structural resistance. All this knowledge could lead in the future to the engineering of metabolic pathways of vascular coating compounds in specific cells, to produce resistant plants against xylem colonizers.

With this article we hope to contribute towards raising awareness of the importance of attaining a better understanding of the structural physico-chemical barriers as a crucial component of resistance towards xylem vascular pathogens. Disease management through host resistance is the most efficient and eco-friendly approach to control pathogens. However, a lot has to be learnt about the complex genetic interactions which govern induced structural resistance in various hosts, to be able to deploy this trait in future cultivars and fight vascular pathogens, agents of the most devastating plant diseases in the field.
